# Microbe–Immune–Stress Interactions Impact Behaviour during Postnatal Development

**DOI:** 10.3390/ijms232315064

**Published:** 2022-12-01

**Authors:** Cassandra Francella, Miranda Green, Giorgia Caspani, Jonathan K. Y. Lai, Kelly C. Rilett, Jane A. Foster

**Affiliations:** 1 Department of Psychiatry and Behavioural Neuroscience, McMaster University, Hamilton, ON L8N 4A6, Canada; 2 Department of Metabolism, Digestion and Reproduction, Imperial College London, London SW7 2BX, UK; 3 The Research Institute at St. Joe’s Hamilton, Hamilton, ON L8N 4A6, Canada; 4 Center for Depression Research and Clinical Care, Department of Psychiatry, University of Texas Southwestern Medical Center, Dallas, TX 75390, USA

**Keywords:** gut-brain axis, microbiome, T cells, behaviour, postnatal development

## Abstract

Decades of research have established the role of microbiota–brain communication in behaviour and brain function. Studies have shown that microbiota composition and diversity are influenced by a variety of factors including host genetics, diet, and other environmental exposures, with implications for the immunological and neurobiological development of the host organism. To further understand early-life interactions between environment, genetic factors, the microbiome and the central nervous system, we investigated the impact of postnatal stress in C57Bl/6 wild type and T-cell deficient mice on microbe–brain interactions and behaviour. Mice were exposed to immune challenge with lipopolysaccharide (LPS) at postnatal day (P) 3 and maternal separation at P9 (16 h overnight). Behavioural assessment of growth and development as well as behaviour (righting reflex, ultrasonic vocalizations in response to brief maternal separation, open field, sociability, and grooming) was conducted. Microbiota diversity and composition of fecal samples collected at P24 revealed reduced alpha diversity in T-cell-deficient mice as well as genotype- and stress-related taxa. Notably, integrated analyses of microbiota and behaviour in the context of immunocompromise revealed key behavioural related taxa that may be important to brain development. These findings are important to determining the influence of genetic and environmental factors on gut microbiota and advances our understanding microbiome–brain signaling pathways on neurodevelopment and behaviour.

## 1. Introduction

The gut microbiota is well-established as a potent modulator of the immune system, and an increasing body of evidence suggests that cross-talk between microbes, environment, and host immunity shapes the maturation and function of the central nervous system (CNS). The precise role of immune signals in connecting the gut ecosystem to CNS development is an important area of study. Recent work has drawn attention to the neonatal microbiome and its developmental trajectory throughout early postnatal life, which parallels ontogenesis of host immunity and proper wiring of neural circuitry in the brain [1]. Indeed, the nature of interactions between these functional systems, especially during critical periods of neurodevelopment, has implications for later life behavioural outcomes and predisposition to neurological disease [1,2].

T cells have recently emerged as key players in the adaptive immune branch of the developing microbiota–gut–brain axis. T-cell development and function are heavily influenced by gut microbes, wherein specific bacterial taxa and their metabolites are known to promote proliferation of distinct T-cell subsets [3,4]. In addition, T cells shape gut-resident microbial communities through a variety of mechanisms, including T-cell-dependent IgA response and activation of antimicrobial peptide (AMP) production [5,6]. Together, these and other bilateral molecular cues not only promote proper maturation of the adaptive immune system but also help facilitate microbial colonization of the neonate gut and establish immune tolerance towards gut commensals.

T cells have also been shown to directly modulate neurodevelopment. The exponential expansion of lymphocyte pools within the first week of life occurs in tandem with hierarchical wiring of neural circuitry in the neonate brain [7]. In adulthood, studies have demonstrated a variety of behavioural abnormalities in immunodeficient models lacking T cells [8,9] as well as the influence of T cells on regulation of social behaviour and synaptic plasticity [7,10]. These modulatory effects are even present prior to birth, as maternal immune activation (MIA) has been shown to impact synaptic development in the offspring via secretion of IL-17a from maternal Th17 lymphocytes [11]. After birth, T cells populate the neonate brain and maintain stable, self-renewing pools in the meningeal space throughout early postnatal life, increasing their potential to modulate the developing neuronal milieu [12]. In this regard, a recent study demonstrated that γδ T cells, the first T-cell subsets to seed peripheral tissues, secrete IL17a to regulate anxiety-like behaviour [13]. Interestingly, this experiment also demonstrated that secretion of IL17a was partially dependent on commensal-derived signals, suggesting an intermediary role of the adaptive immune system in transmitting microbial signals to the brain. Finally, T-cell-derived IFN-γ, which displays significantly reduced levels in germ-free mice, has been shown to alter the balance between excitatory and inhibitory synapse formation in favour of inhibitory signalling [14]. Together, these results suggest T cells and a variety of T-cell-derived signalling molecules can directly and indirectly impact wiring of the developing brain. This has been corroborated in recent experiments that have reintroduced T cells in the early-life window of immunodeficient mice, an intervention that not only remediates behavioural abnormalities in adulthood but partially rescues cortical hyperconnectivity observed in untreated controls [15].

In parallel with these observations, T-cell-deficient (*TCRβ-/-δ-/-)* mice display abnormalities in both anxiety-like behaviour [16] and hypothalamic pituitary axis (HPA)-mediated stress response [17]. These behavioural changes were accompanied by a genotype-specific microbiome and metabolite signature, which was coincident with altered levels of neuroactive metabolites in the brain [18]. Importantly, the latter experiments took a longitudinal approach that revealed a marked difference in the developmental trajectory of microbiome and metabolome in *TCRβ-/-δ-/-* mice compared to WT controls, suggesting a temporal impact of T-cell signalling on both microbes and brain chemistry.

In light of these results, this study sought to further explore interactions between T cells, microbes, and postnatal environment on the developing brain. To achieve this, both wild type B6 and *TCRβ-/-δ-/-* mice were exposed to two environmental stressors, namely maternal separation (MS) and postnatal immune challenge (LPS), within the first two weeks of life. In parallel, developmental milestones in behaviour were assessed using our developmental milestones behavioural (DMB) pipeline from postnatal day 4 (P4) to P28, as well as microbiome profiling at P24 (postweaning) [19]. There is currently a large body evidence to suggest that both the microbiome [20,21] and behavioural outcomes [21,22] are impacted by stress in critical developmental windows. Given the importance of immune signalling in gut–brain axis development, this study tested the hypothesis that early-life stress has different impacts that depend on the immune status of the host. Together, this experiment advances our understanding of the role of the immune system and immune–microbe interactions in early life as well as the role of environmental stressors in modulating critical windows of neurodevelopment.

## 2. Results

### 2.1. T-Cell-Deficient Mice Showed Altered Postnatal Behaviour in Comparison to B6 Mice

**Figure 1:**
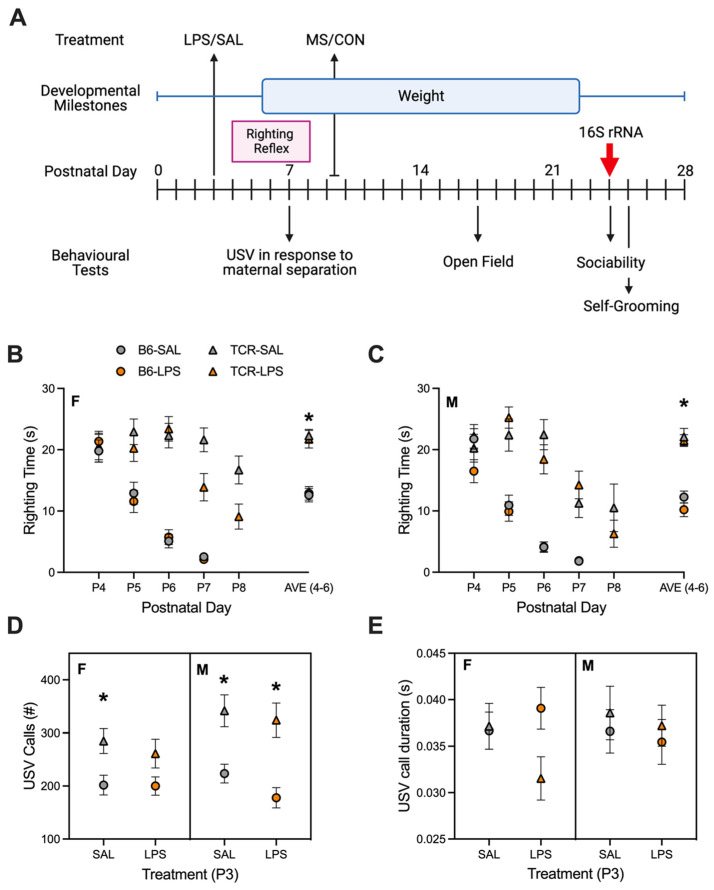
Postnatal behaviour differed in T-cell deficient mice. (**A**) Experimental design showing postnatal challenges, developmental outcomes, and behavioural tests used in the first 4 weeks of postnatal life. Red arrow indicates date of fecal sample collection for 16S rRNA sequencing analysis for microbiota. (**B**) Daily righting reflex time and P4–6 average time (s) for female mice. (**C**) Daily righting reflex time and P4–6 average time (s) for male mice. (**D**) Number of USV calls during 3 min separation from dam for male and female mice. (**E**) Average USV call duration during 3 min separation from dam from male and female mice. * *p* < 0.05, mean ± S.E. provided. Panel A was created using Biorender.

The DMB pipeline shown in Figure 1 was used to compare early life development in B6 and *TCRβ-/-δ-/-* mice. A developmental delay in righting reflex was observed in *TCRβ-/-δ-/-* mice. As shown in Figure 1B (females) and Figure 1C (males), the righting reflex in both female and male B6 mice developed between P4 and P6, visualized as a daily reduction in righting time, whereas the righting reflex in *TCRβ-/-δ-/-* mice developed between P6 and P8 (main effect of genotype—F(1,267) = 587.8, *p* < 0.0001). The average righting time (P4–6) in B6 mice was significantly lower than that of *TCRβ-/-δ-/-* mice (*p* < 0.05). No differences were observed between male and female mice within each genotype, and there was no effect of P3 immune challenge (LPS) on righting reflex (*p* > 0.05). A main effect of genotype on USV calls was observed (F(1,272) = 654,380.9, *p* < 0.0001). *TCRβ-/-δ-/-* mice had an increased number of USV calls in response to maternal separation compared to B6 mice (Figure 1D), with male *TCRβ-/-δ-/-* mice showing a significantly higher number of USV calls than male B6 mice. USV call duration did not differ between groups (Figure 1E).

**Figure 2:**
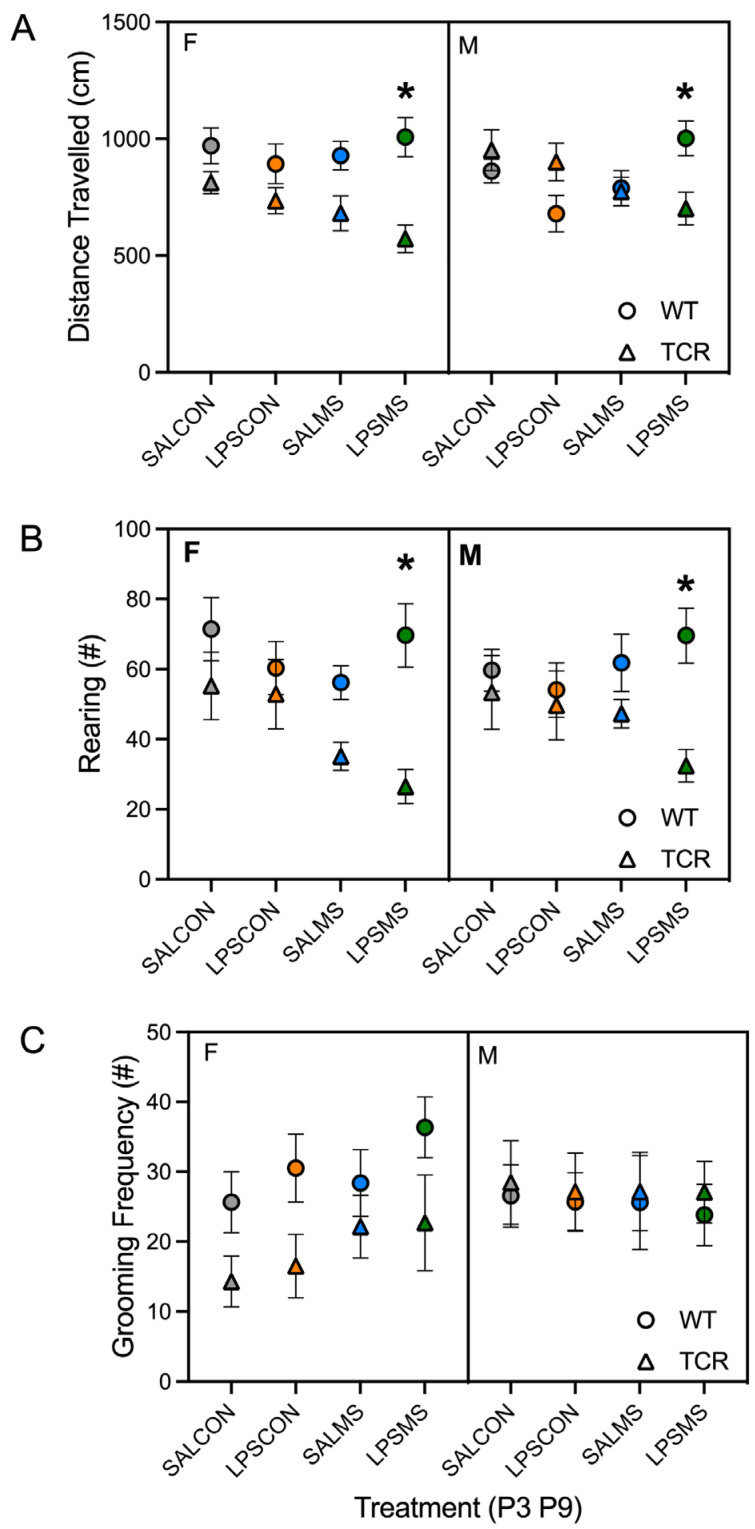
Behavioural comparison of wild-type (WT) and T-cell-deficient (*TCRβ-/-δ-/-*) mice. Distance travelled (**A**) and rearing (**B**) measured in the open-field test at postnatal day 17 (P17) in female (F) and male (M) mice for different treatment conditions at P3 and P9—SALCON, SALMS, LPSCON, and LPSMS. Self-grooming was tested at P25 (**C**) * *p* < 0.05, mean ±S.E. provided, SAL, saline; LPS, lipopolysaccharide; CON, control; MS, maternal separation.

Analysis of open-field behaviour on P17 revealed a main effect of genotype (F(1,273) = 12.2, *p* = 0.001) on total distance travelled as well as a significant interaction between genotype and sex (F(1,273) = 12.1, *p* = 0.001) and between genotype and treatment (F(1,273) = 5.16, *p* = 0.002). Both female and male *TCRβ-/-δ-/-* in the LPSMS treatment group showed reduced distance travelled in the open field relative to the B6 counterparts (Figure 2A). No other differences were observed in distanced travelled.

**Figure 3:**
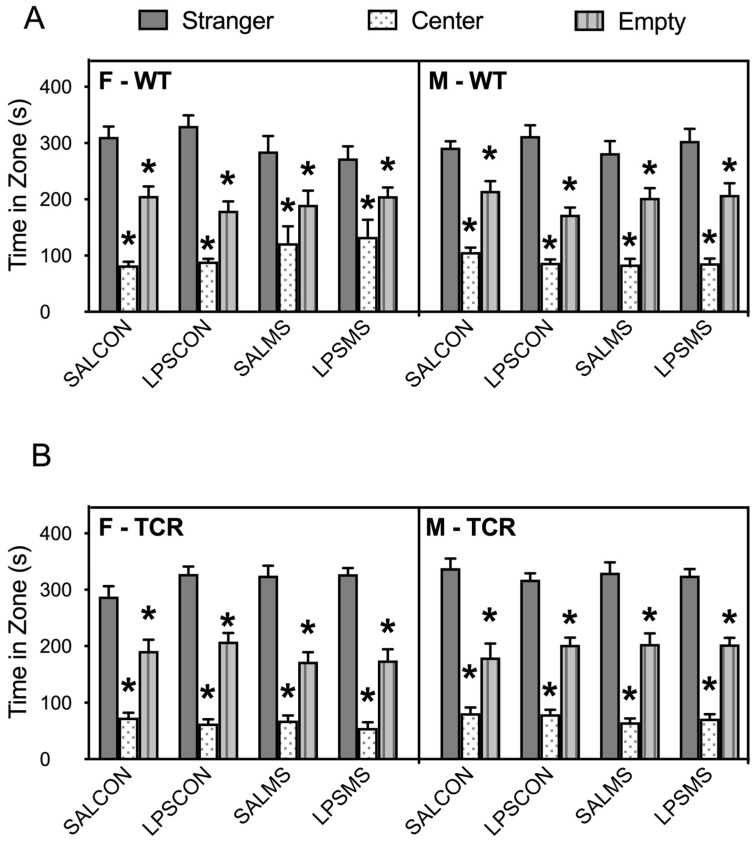
Social behaviour tested in three-chamber test at postnatal day 24. Wild-type (WT) (**A**) and T-cell-deficient (*TCRβ-/-δ-/-*) (**B**) showed typical sociability measured as social preference for chamber with stranger mouse. * *p* < 0.05, significantly different from stranger time.

Analysis of rearing in the open field revealed a main effect of genotype (F(1,273) = 20.8, *p* < 0.0001) and a significant interaction between genotype and treatment (F(1,273) = 3.84, *p* = 0.01). Similar to total distance travelled, female and male *TCRβ-/-δ-/-* mice in the LPSMS treatment group showed reduced rearing in the open field compared to B6 (Figure 2B, *p* < 0.05)). No difference in grooming behaviour was observed between B6 and *TCRβ-/-δ-/-* mice (Figure 2C), and male and female B6 and *TCRβ-/-δ-/-* mice displayed normal sociability at P24 (Figure 3).

### 2.2. Microbial Diversity and Compositional Differences between B6 and TCRβ-/-δ-/-

**Figure 4:**
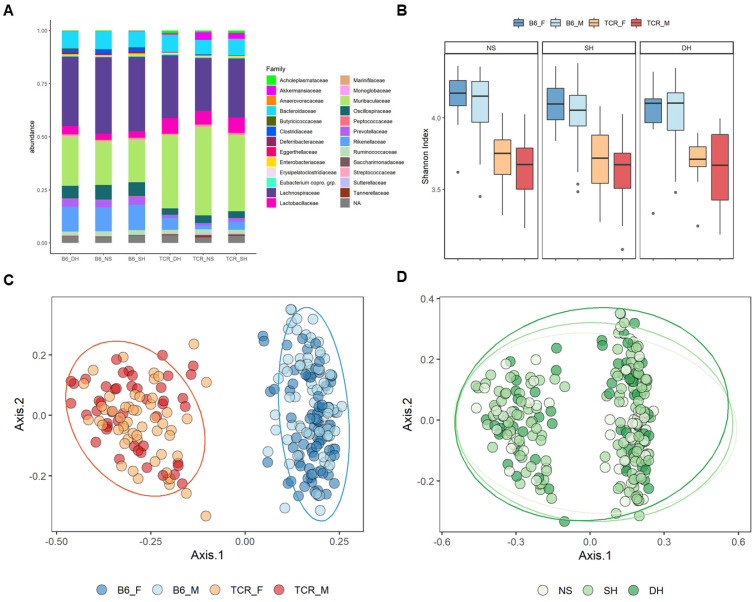
Gut microbiome characteristics vary between T-cell-deficient and WT mice. (**A**) Average relative abundances of recognized bacterial families based on 16S sequence data between experimental groups (genotype, sex, and stress condition). (**B**) Alpha diversity plots showing decreased Shannon index in T-cell-deficient mice compared to controls (**C**,**D**) and beta diversity plots to visualize compositional in the microbiota between the T-cell-deficient (orange) and WT (blue) mice. PCoA plot of Bray–Curtis index (**C**) coloured by genotype and sex and (**D**) coloured by stress condition, highlighting the dominant effect of genotype on microbiome structure.

Fecal samples were analyzed by 16s rRNA gene sequencing to assess their microbial diversity and compositional differences. Prior to filtering, 2843 unique ampicon sequence variants (ASVs) were present in the dataset with a maximum read count of 217,474 and minimum reads of 3355 per sample. Median number of reads per samples was 75,543. Differences in both alpha and beta diversity between genotypes were observed (Figure 4). *TCRβ-/-δ-/-* mice exhibited a decrease in alpha diversity compared to B6 mice (Chao1 < 0.05 for all rarefaction depths and Shannon index < 0.05 between genotypes). In addition, *TCRβ-/-δ-/-* and B6 mice clustered within genotype, with PERMANOVA revealed significant clustering by genotype for both NMDS and PCoA plots (Figure 4, PERMANOVA *p* < 0.05). Compositional differences were detected between *TCRβ-/-δ-/-* and B6 mice; however, no significant differences in beta diversity or beta dispersion were observed between stress condition groups (*p* < 0.05, Figure 4).

### 2.3. Differential Taxonomic Abundance between B6 and TCRβ-/-δ-/-

Out of 2843 initial taxa, 207 ASVs met initial prevalence/abundance criteria and were retained in the analytical dataset. ASVs that did not meet criteria were agglomerated to 317 genus-level taxa using the *tax_glom()* function. Twenty-nine glommed taxa met criteria in the second round of filtering and were added to the analytical dataset. The remaining 288 taxa were agglomerated into a single “other” category, resulting in a final dataset included a total of 237 taxa.

**Figure 5:**
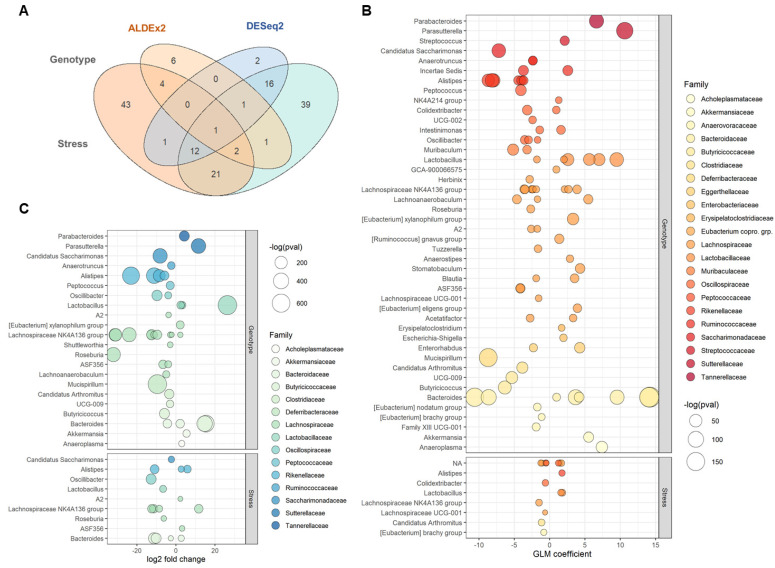
Differential abundance (DA) analysis reveals taxa that vary significantly between genotypes and stress conditions. (**A**) Venn diagram of consensus analysis between multiple DA analysis methods (DESeq2 and ALDEx2) showing overlap between significantly variable taxa across stress conditions (top) and genotypes (bottom). (**B**,**C**) Differentially abundant taxa significant to a 0.05 level using ALDEx2 (orange) and DESeq2 (blue) between genotype groups (top) and stress conditions (bottom). Positive log_2_fold change/GLM coefficient indicates higher abundance in *TCRβ-/-δ-/-* mice compared to B6 controls and vice versa for negative fold changes/coefficients.

Negative binomial regression (DESeq2) revealed 93 ASVs that were significantly differential expressed between the genotypes at the genus level. Of those, 57 ASVs exhibited a negative log_2_fold change, indicating lower relative abundance in *TCRβ-/-δ-/-* mice compared to B6 and 39 ASVs with a positive log_2_fold change, indicative of higher relative abundance in *TCRβ-/-δ-/-* mice compared to B6 (Figure 5). These T-cell-related taxa are listed in Supplementary File S1. The majority thereof belonged to the families *Muribaculaceae* and *Lachnospiraceae*, which together comprised 54 of the 93 taxa showing significant changes between genotypes. Within these families, all *Lachnospiraceae* taxa were significantly more abundant or exclusively present in *TCRβ-/-δ-/-* mice while displaying low or undetectable levels in B6 controls. These taxa were mostly identifiable to the genus level, with the majority belonging to the *Lachnospiraceae* NK4A136 group and the remainder distributed amongst genera including *Blautia*, *Roseburia*, and ASF356. By contrast, taxa within the *Muribaculaceae* family were mostly unidentified at the genus level and displayed a range of log_2_fold change values, with some far more abundant in B6 compared to *TCRβ-/-δ-/-* mice and vice versa. Similar results were obtained using ALDEx2, which returned 169 differentially abundant taxa across genotypes (Figure 5, Supplemental File S1). Out of these sets, 80 taxa were mutually significant across methods, including 29 *Muribaculaceae* ASVs and 12 of the aforementioned *Lachnospiraceae* taxa (Figure 5, Supplemental Figure S2, Supplemental File S2). Other notable taxa that increased in *TCRβ-/-δ-/-* mice based on our consensus approach include *Akkermansia*, *Anaeroplasma*, and some strains of *Bacteroides*, while B6 mice showed an increase in other taxa belonging to this genus. Otherwise, B6 controls also harboured larger populations of the genera *Lactobacillus*, *Alistipes*, and *Peptococcus* relative to their *TCRβ-/-δ-/-* counterparts.

Stress was found to have a much more subtle effect on microbiome composition as compared to genotype, wherein ALDEx2 and DESeq2 only identified 18 and 33 taxa that were significantly impacted by stress condition, respectively (listed in Supplementary File S1). Only two of these taxa survived consensus filtering: *Eubacterium coprostanoligenes* group sp139 and *Lactobacillus reuteri* sp169. Interestingly, when examining the effects of genotype–stress interaction on ASV abundance, seven mutual taxa returned significant results between methods, including some unidentified *Lachnospiraceae* and *Muribaculaceae* as well as *Lactobacillus reuteri*, *Akkermansia muciniphila*, and *Candidatus arthromitus* (Figure 5, Supplemental Figure S2, Supplemental File S2).

### 2.4. Microbe–Behaviour Relationships in B6 and TCRβ-/-δ-/- Mice

**Figure 6:**
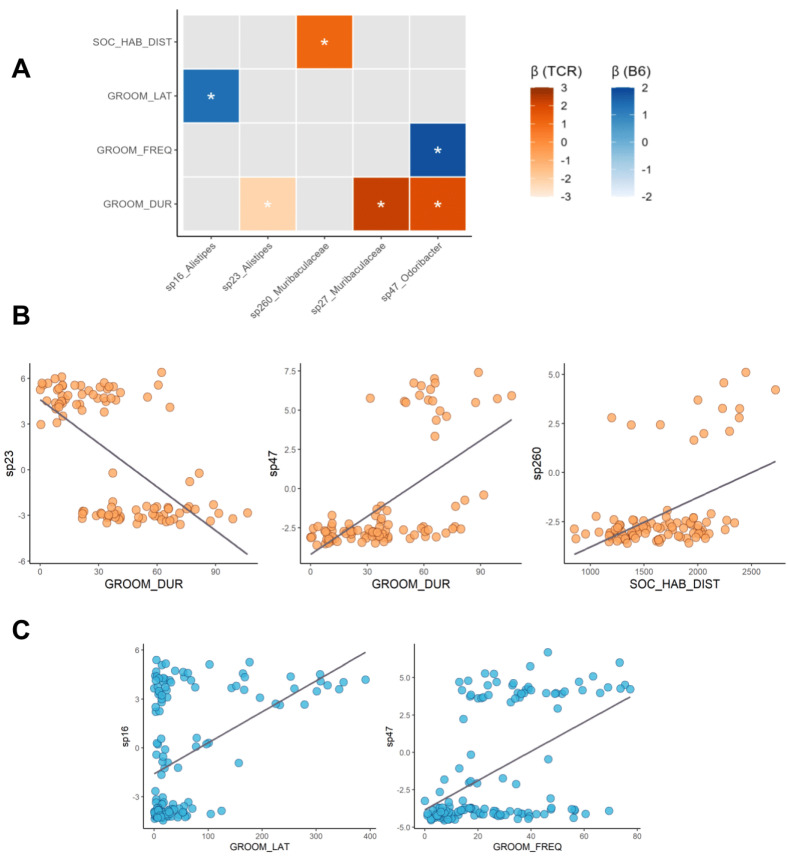
MaAsLin2 significant results and associations between behavioural measures (open field, social behaviour, and self-grooming) and gut microbiota composition at the ASV level of *TCRβ-/-δ-/-* and B6 mice at postnatal day 24 (P24). (**A**) Color scale-bar shows scaled correlations between taxa and factors, ranging from the highest to lowest for each genotype. Stars indicate significance at FDR < 0.05. (**B**,**C**) Exemplary correlations between individual ASV abundances and behavioural metrics for B6 (blue) and *TCRβ-/-δ-/-* (orange) mice.

To explore relationships between behavioural measures and microbiome composition, we tested for multivariable associations between phenotype and retain-resolved microbial taxa using MaAsLin2. Tests were conducted independently within each genotype group to account for large differences in microbiome structure. Overall, grooming duration, social habituation distance, grooming latency, and grooming frequency were found to have significant associations with specific bacterial taxa, with the former two behaviours only showing correlation in *TCRβ-/-δ-/-* mice and the latter two only in B6 (Figure 6). All associations reached a high level of significance (q < 0.05). In *TCRβ-/-δ-/-* mice, *Rikenellaceae alistipes* sp23 was negatively correlated to grooming duration, while this behaviour displayed a positive relationship with *Muribaculaceae* sp27 and *Marinifilaceae odoribacter* sp47. *Muribaculaceae* sp260 was also weakly (positively) correlated to social habituation distance (distance travelled during the 5 min habituation period). In B6 mice, *Rikenellaceae alistipes* sp16 and *Marinifilaceae odoribacter* sp47 were positively correlated to grooming latency and grooming frequency. An omnibus analysis that incorporated genotype as an additional fixed effect, a number of associations were observed between microbiome features and social or grooming behaviour (Supplemental Figure S3). These included various *Lactobacillus* and *Alistipes* strains as well as unidentified taxa belonging to the families *Lachnospiraceae* and *Muribaculaceae*.

## 3. Discussion

In the present study, developmental milestones were assessed using a developmental milestones behavioural (DMB) pipeline and the impact of genetic risk factors and early-life events on the trajectory of behavioural development was investigated. Using a double-hit model of early-life stress (immune challenge (LPS) and maternal separation (MS)), results demonstrated that immune compromise (i.e., T-cell deficiency) increased stress reactivity in different behavioural readouts during postnatal development: ultrasonic vocalizations at P7 and locomotion (open field) at P17. The absence of T cells led to lower microbial diversity and altered the gut microbiome composition post weaning, characterized by a loss of butyrate-producing taxa. Notably, stress significantly impacted the abundance of select taxa independently of genotype. A number of taxa were significantly impacted by a genotype–stress interaction, demonstrating an impact of host genetic background on stress response. Finally, in both omnibus- and genotype-partitioned multivariable association analysis, multiple ASVs were significantly correlated to behavioural traits, suggesting a link between specific microbes and behavioural outcomes that result from microbiota–immune interactions.

In immunocompetent mice, neonatal exposure to LPS has been shown to induce gene expression changes in corticotropin-releasing hormone (CRH), CRH receptors, and the major corticosteroid receptors in the hippocampus and hypothalamus [23], with sex-specific effects on exploratory and anxiety-like behaviours [24]. These studies suggest that early-life immune activation alone has a significant impact on physiological and behavioural readouts of stress reactivity in later life. The application of our behavioural pipeline revealed that, compared to B6 mice, immunocompromised mice exhibited neurodevelopmental delays as assessed by the righting reflex as well as an exaggerated behavioural response to stress. The latter was evidenced by as an increase in ultrasonic vocalizations (USVs) observed at P7 in the LPSMS *TCRβ-/-δ-/-* group (in males only) and at P17 as a decrease in exploratory behaviour in male and female *TCRβ-/-δ-/-* in the LPSMS group. Previous studies have shown that a single maternal separation of pups from the dam on P9 leads to long-term changes in behaviour in the brain although studies to date have examined only outcomes in adult mice. For example, MS on P9 resulted in a depressive-like phenotype in adulthood 129S1/SvlmJ mice but not in other inbred strains [25], increased anxiety-like behaviour and decreased spatial learning in adult Balb/C mice [26], as well as increased sensitivity to an immune challenge in adulthood [26]. In addition, MS on P9 in *microtubule-associated protein-6* heterozygous mice resulted in spatial deficits and neuroanatomical changes [27]. Our developmental findings are consistent with previous work in adult mice showing increased plasma corticosterone at baseline and an exaggerated increase in stress-related gene expression in the hypothalamus and central amygdala after repeated restraint stress in *TCRβ-/-δ-/-* relative to B6 mice [17]. Moreover, these results are consistent with reported brain volumetric changes in stress-related brain regions such as the hippocampus, hypothalamus, amygdala, and bed nucleus of the stria terminalis in *TCRβ-/-δ-/-* mice, indicating an overall sensitivity of these circuits to immune deficiency [16]. Together, these data suggest that immune status of the host is a key determinant in the adaptive response to environmental stress, wherein behavioural outcomes in adulthood were modulated by early-life stressors. Furthermore, the observed behavioural differences at 1 week of age demonstrate that the effects of stress can interact with host immunity in a very early developmental window, highlighting the importance of a longitudinal behavioural pipeline that accounts for a range of postnatal epochs.

The notion that germ-free (GF) rodents share many of the molecular (e.g., elevated hypothalamic CRH expression [28]) neuroanatomical (altered brain volume [29]), and behavioural features (reduced anxiety ([30,31]) observed in T-cell-deficient mice suggests a role for microbe–immune interactions in shaping stress sensitivity. In order to further explore this concept, we compared 16S rRNA sequencing results from fecal samples of *TCRβ-/-δ-/-* and B6 mice. Compared to B6 mice, *TCRβ-/-δ-/-* mice displayed a less diverse and compositionally distinct microbiota, as demonstrated by reduced alpha diversity and strong strain-based clustering in beta diversity ordination space. This is consistent with previous work conducted in *TCRβ-/-δ-/-* mice [18] as well as in other T-cell-specific immunodeficiency models [32,33], demonstrating a consistent relationship between host immunity and microbiome architecture. Furthermore, we identified a key set of microbial taxa that define the microbiome signature of *TCRβ-/-δ-/-* mice. The microbiome of *TCRβ-/-δ-/-* mice was characterized by a clear loss of butyrate-producing bacteria, such as *Lachnospiraceae NK4A136 group*, *Anaerotruncus*, *Oscillibacter*, *Butyricicoccus*, and *Roseburia*. Previous metabolomic analysis of cecal and colonic tissue collected from B6 and *TCRβ-/-δ-/-* mice reported significantly lower abundance of cecal and fecal butyrate in the immunocompromised group [18], supporting a role for T cells in modulating butyrate concentrations via a restructuring of the gut microbial community. The short-chain fatty acid (SCFA) butyrate is a common colonic metabolite produced through the fermentation of indigestible fiber by the gut microbiota. Butyrate exerts protective effects by modulating barrier integrity [34,35] and mucus production [36] and contributes to the maintenance of an anaerobic environment in the large intestine [37]. In addition to its role as the main energy source for colonocytes, butyrate has both local and systemic immunomodulatory properties. Notably, butyrate acts as a histone deacetylase (HDAC) inhibitor to promote the differentiation of CD4+ naïve T cells into colonic regulatory T cells (Tregs), contributing to a protective, tolerogenic phenotype [38,39,40]. The results of the present study demonstrate the bidirectional nature of microbe–immune interaction in modulating the composition of the gut microbiota. Future research should explore the mechanistic underpinnings of this communication loop and explore the hypothesis that feedback signals from gut-resident T cells may skew microbiome composition and functional output towards anti-inflammatory mediators (e.g., butyrate) that promote immune regulation.

*TCRβ-/-δ-/-* mice exhibited significantly higher abundance of *Akkermansia muciniphila* and several species belonging to the *Lactobacillus* genus, notably *Lactobacillus reuteri* and *Lactobacillus johnsonii*, which aligns with previous observations [18]. *A. muciniphila* has been identified as potent inducers of antigen-specific IgA and IgG1 responses via a mechanism mediated by T follicular helper cells [41]. A lack of this T-cell-dependent humoral response and the resulting absence of microbial antigen-directed antibodies may explain the expansion of these taxa in T-cell-deficient mice. By contrast, *Lactobacillus* can play a supportive role in immunomodulation, producing compounds such as lactate and acetate that cross-feed butyrate and propionate-producing strains [42]. Specific strains of *Lactobacillus* have also been shown to enhance apical expression of *monocarboxylate transformer 1* (*MCT1*), a key butyrate transporter, and thus increase colonic SCFA absorption [43]. It is thus interesting that these strains were increased in the context of T-cell deficiency, suggesting a different role for *Lactobacillus* in the ecosystem structure in a niche with reduced butyrate-producing metabolic capacity.

Interestingly, *L. reuteri* and *L. johnsonii* have been shown to display potent immunomodulatory properties in their own right, stimulating various components of the innate immune system such as dendritic cells [44], macrophages [45], and toll-like-receptor (TLR)-mediated immune response [46,47,48]. In an immunosufficient context, this has downstream effects on biasing the differentiation of anti-inflammatory T-cell subsets [49,50] as well as intraepithelial leukocytes (IELs) derived from lamina propria CD4+ cells [51]. However, in absence of functional T cells and a coordinated adaptive immune response, it is possible that increased stimulation of the innate immune system may constitute a form of compensation.

Despite a lack of large compositional differences in the microbiome of stressed versus non-stressed mice, a small number of ASVs were identified that were sensitive to both immune compromise and stress, suggesting that T-cell deficiency influences the effect of stress on specific microbial communities. In this regard, stress is well known to impact the microbiome and vice versa. For one, environmental stress has been shown to reduce richness and functional diversity of the microbiota [52], while stress-related catecholamines and other neuroendocrine hormones secreted by cells in the gastrointestinal (GI) tract can directly modulate microbial growth [53]. Further, signalling via the vagus nerve and enteric nervous system alter GI motility and reduce digestive activity, impacting the gut microbiota through changes in their physical environment and biochemical milieu [54]. Exposure to environmental stress has been shown to reduce the bacterial production of SCFAs and expression of their receptors, implicating stress as a potent modulator of microbiome functional output [55]. Moreover, mice exposed to restraint stress showed drastic changes in microbial community structure, including a decrease in abundance of *Lactobacillus* and significantly reduced SCFA production [56]. These changes in the microbial ecosystem are often accompanied by increased immune activation and can be linked to changes in anxiety and depressive-like behaviours [57] in a dynamic host–microbe cycle that can be modulated by the presence of stressful stimuli.

In this experiment, consensus analysis revealed that *Eubacterium coprostanoligenes group* sp139 was depleted as a result of stress, while abundance of *Lactobacillus reuteri* sp169 was increased. Interestingly, when examining the interaction effect between stress and genotype, eight taxa were significantly impacted, including *A. muciniphila*, *Candidatus Arthromitus*, unidentified *Muribaculaceae* and *Lachnospiraceae*, as well as *L. reuteri*. Of particular note is the apparently large increase in *L. reuteri* between stressed and unstressed mice, which is in direct conflict with previous studies that have shown notable stress-induced reductions in *L. reuteri* populations [58]. However, it is important to note that this effect was observed in stressor-exposed CD-1 mice but not in inbred C57BL/6 mice. For this study, it appears that our results are largely driven by changes in microbial populations in *TCRβ-/-δ-/-* mice rather than B6 controls. Indeed, examination of relative abundance data reveals a significant increase in *L. reuteri* in stressed immunodeficient animals, whereas the low abundance of this taxon in B6 mice obscured any obvious change. This is notable, as it indicates that not only host genetic background but also host immune status can modify the impacts of stress on this specific strain, and removal of adaptive immune components was sufficient to not only mitigate but completely invert this effect. In addition to *L. reuteri*, as aforementioned, the other strains affected by stress–genotype interaction have fascinating immunomodulatory properties and occupy a range of ecological niches within the gut ecosystem. Future experiments could seek to understand the mechanistic underpinnings of how stress interacts with the microbiome in the presence or absence of host adaptive immunity such that key bacterial taxa may gain or lose a competitive advantage through immune manipulation.

The final component of our analysis sought to explore relationships between behavioural measures and microbial taxa using a multivariate association model (MaAsLin2). We found multiple associations between grooming behaviours and taxa from the *Odoribacter* and *Alistipes* genera as well as two unidentified *Muribaculaceae* taxa. *Odoribacter* is a somewhat pleotropic microbe: while it is a known butyrate producer [59], it has also been suggested as an opportunistic pathogen and displays increased abundance in response to high-fat feeding [60]. *Alistipes*, another member of the *Bacteroidetes* phylum, is known to have a range of implications for behaviour and mental health, with an apparent involvement of this genus in depression and neurodevelopmental disorders [61]. Finally, some studies have linked *Muribaculaceae* to depressive-like [62] and autism-like behaviours [63]; however, the largely uncharacterized nature of this family and the inconsistent relationships between various constituent ASVs and phenotypic markers limits the interpretability of such results. Interestingly, when conducting the same analysis across all samples with genotype as an additional fixed effect in the model, additional relationships emerged between microbes and grooming and social behaviour. Notably, multiple ASVs representing *L. reuteri* were strongly correlated with distance travelled during social habituation. This observation aligns with previous work demonstrating that *L. reuteri* administration rescues social deficits in genetic, environmental, and idiopathic mouse models of ASD [64,65] and provides evidence that the effects of *L. reuteri* may emerge independently of other host genetic factors.

## 4. Materials and Methods

### 4.1. Animals and Experimental Design

Male and female T-cell receptor (TCR) double-knock-out mice, lacking both the *β* and the *δ* chain of the TCR (*TCRβ-/-δ-/-*), and wild-type C57BL/6 (B6) mice were bred in house at St. Joseph’s Healthcare animal facility. Mice were maintained under a 12 h light–12 h dark cycle, with lights on at 5 AM and access to food and water ad libitum. Birth was set as postnatal day 0 (P0), and on P2, litters were culled to 10 pups and pups were uniquely tattooed on their paw for identification. The experimental design is provided in Figure 1. On P3, pups were administered 0.1 mg/kg of lipopolysaccharide (LPS) in 50 µL/g of saline or vehicle (SAL). On P9, pups were maternal separated (MS) from the dam overnight (16 h) or left with dam (CON). On P21, pups were weaned and caged by sex with up to 4 littermates per cage. Following behavioural testing on P24, fecal samples were collected from each mouse and were frozen on dry ice immediately and stored at −80 °C until being sent for 16s rRNA gene sequencing at McMaster University’s Genome Center. All experiments were completed in accordance with the guidelines set out by the Canadian Council on Animal Care and were approved by the McMaster Animal Research Ethics Board in Hamilton, Ontario, Canada.

### 4.2. Behavioural Testing

#### 4.2.1. Righting Reflex

On P4–8, pups were tested for motor development by timing their ability to right themselves after being placed on their backs. A completed righting was defined by all four paws on the ground simultaneously. Prior to testing, the dam was removed from the home cage, and each pup was tested in turn. The maximum separation time from the dam was 10 min. Righting reflex (RR) was completed at 4 PM. Righting time was measured with a stopwatch; if the pup was not able to right itself by 30 s, the pup was manually righted, and righting time recorded as 30 s.

#### 4.2.2. USV Recordings

On P7, pups were consecutively maternally separated and placed in a custom-made sound-attenuating chamber. Testing took place during the first half of the active period, at least one hour after the active cycle began. Ultrasonic vocalizations (USV) were recorded for 3 min, and then, the pup was transferred to a different heated holding cage. After all pups in a litter were recorded, the pups were returned to the dam. Vocalizations were digitized in real time using an Avisoft UltraSoundGate 116–200 recording device, and USG CM116/CMPA microphone, and subsequently analyzed with Avisoft SAS Lab Pro. The microphone was clamped to a retort stand and situated 17.5 cm above the centre of the recording chamber.

#### 4.2.3. Open Field

At P17, pups were tested in the open field. Behavioural testing was conducted in a non-colony room after a 30 min habituation to the room. After habituation, the dam was removed from the litter. Testing took place in low light (200 lux) during the first half of the active period. Behaviours were automatically recorded for 15 min using the Kinder Scientific Smart Rack System consisting of a 24 cm wide × 45 cm long × 24 cm high cage rack system, with 22 infrared beams (7 X and 15 Y) and a rearing option (22 additional beams). A Plexiglas^®^ box was placed at one end of the chamber to reduce the testing chamber size to 24 × 23 cm. Data were collected using MotorMonitor^®^ software (Kinder Scientific, Poway, CA, USA). Up to 6 pups were tested at a time in separate chambers. After all pups in a litter had undergone testing, they were returned to the dam; maternal separation did not exceed 30 min.

#### 4.2.4. Sociability

At P24, sociability was tested using a 3-chamber apparatus [14] in a non-colony after 20 min habituation to the room. Mice were handled for 2 min each on the two days leading up to sociability test. Testing took place during the first half of the active period. Mice were acclimatized in the centre zone of the apparatus for 5 min. Subsequently, an age- and sex-matched stranger mouse was placed in an inverted cup in one of the side chambers and an empty inverted cup in the other side chamber. The test mouse was able to access the entire apparatus for 10 min. Live-tracking and videotape analysis was conducted using EthoVision ^®^ XT 8.5 software.

#### 4.2.5. Self-Grooming

At P25, spontaneous self-grooming was measured. Mice were acclimatized to an empty cage for 10 min. Mice were then video recorded for 10 min [66].

#### 4.2.6. Behavioural Data Analysis

Analysis of behavioural data to identify main effects of genotype, sex, and treatment was completed in SPSS Ver. 25 by multivariate general linear model, followed by Bonferroni post hoc tests. Omnibus analysis was conducted in behavioural data prior to P9 (RR and USV) that included 2 treatment (P3) groups (LPS and SAL) and in behavioural data following P9 (open field, sociability, self-grooming) that included 4 treatment (P3_P9) groups (SALCON, SALMS, LPSCON, and LPSMS). Behavioural data were visualized using GraphPad Prism ver 9.4.1.

### 4.3. 16S rRNA Gene Sequencing and Amplicon Sequence Variant (ASV) Processing

DNA was extracted from the fecal samples and the sequences from the gene variable 3 (v3) region of the 16s rRNA gene was amplified and sequenced using the Illumina MiSeq platform [67] at McMaster University’s Genome Center. The raw FASTQ files from the Illumina were then processed through DADA2, a Bioconductor package [68,69]. The sequences were truncated, the PCR primers were trimmed off, and expected errors were discarded. Through the function *learnErrors*, estimation of the error rates of the sequences occurred. The filtered sequences were then dereplicated using *derepFastq* function, and unique sequences were generated. Amplicon sequence variants (ASVs) were generated from the sequences using the *dada* function. Forward and reverse reads were done independently. Using the *mergePairs* function, reverse and forward reads were merged to refine ASVs, in which a count table was then generated. Chimeric sequences were removed using the *removeBimeraDenovo* function [68,69]. Finally, taxonomy was assigned up to the genus level using the Ribosomal Database Project (RDP) classifier and Silvia reference database (version 1.3.8). Using the *phyloseq* package (ver 1.40.0) in R, singleton ASVs were removed, and the final ASV table was generated for further analysis.

### 4.4. 16S rRNA Data Statistical Analysis

#### 4.4.1. QC of Sequencing Data

Host sequences were removed from the ASV table to include only bacterial sequences. In addition, ASVs that exhibited a mean of less than 10 were also removed. The same filtering was performed for relative abundance data. Principle component analysis (PCoA) for Beta-diversity using raw ASV data revealed three individual clusters: two B6 clusters and one *TCRβ-/-δ-/-* cluster (Supplementary Figure S1). Demographic factors including reads, litter, and seasonal time of birth were analyzed for each cluster using the Shannon index to investigate the explanation of these clusters. Graphs were created and visualized in GraphPad Prism, Version 8. Analyses revealed that the subset of B6 mice exhibited a low Shannon diversity of less than 2.0 as well as exhibited litter effects (Supplementary Table S1). Due to these findings, these mice were removed from subsequent analyses.

#### 4.4.2. Alpha Diversity Analysis

Diversity analyses were completed within R version 3.4.3. Alpha diversity was calculated using the raw ASV data. To observe differences in sequencing depth between samples, rarefaction curves were produced. Individual samples were rarefied 10 times at multiplied depths up to the minimum sequencing depth. The mean diversity at each sampling depth was taken and visualized using a rarefaction curve, which looked at differences in sequencing depth both within sex for each genotype and between genotypes. This was graphed within Graph Pad, Prism version 9. Kruskal–Wallis test was used to compare differences between groups, and a *p*-value of <0.05 was determined as statistically significant. The Benjamini–Hochberg (BH) procedure was used to correct for FDR. Alpha diversity metrics Shannon and Chao1 index were used in the analysis.

#### 4.4.3. Beta Diversity Analysis

Beta diversity analysis was calculated using raw ASV data. Principle coordinate analysis (PCoA) as well as non-metric dimensional scaling (NMDS) were generated to analyze the microbial differences between the samples. Bray–Curtis distance metric was included in the analysis. For the NMDS plot, 95% confident ellipses were calculated with the stat-ellipse function within the *ggplot2* package. To the assess the influence of genotype and sex on beta diversity, a permutational multivariate analysis of variance (PERMANOVA) was conducted using the distance metrices function *adonis()* from the *phyloseq* R package.

#### 4.4.4. Retain-Resolve Preprocessing of Microbiome Data

Criteria for ASV retention was set to prevalence > 50% or mean relative abundance > 0.1% and prevalence > 10%, with a minimum read count of 10 required to constitute true detection to reduce overdispersion. ASVs that did not meet criteria were agglomerated to the genus level using the *tax_glom()* function from the *phyloseq* package, retaining taxa that had assignments missing (NA) at the genus level. Resolved taxa were then filtered by the same criteria: prevalence > 50% or mean relative abundance > 0.1% and prevalence > 10% with a detection threshold of 10 reads. Finally, to retain integrity of the relative abundance data, the remaining taxa were agglomerated into a single “other” category (https://rdrr.io/github/SarahAsbury/retainresolve (accessed on 30 October 2022)).

#### 4.4.5. Bacterial Abundance Analysis: Assessing Differences between B6 and *TCRβ-/-δ-/-* Mice

Differential abundance was assessed by constructing a model using both DESeq2 and ALDEx2 on retain-resolved microbiome data to determine the effect of genotype on ASV abundance while accounting for treatment condition and genotype–treatment interaction in a multi-factor design. For the purposes of model construction, genotype was encoded as two levels (B6, TCR), while stress was encoded as three conditions: no stress (NS), single hit (SH, either MS or LPS), or double hit (DH, both LPS and MS). DESeq2 utilizes a negative binomial distribution and adjusts for size factors, normalizing for differences within sequencing depth between samples. This regression used the default settings but without independent filtering. The output of these results was expressed as log_2_fold change in *TCRβ-/-δ-/-* mice relative to B6. Contrasts of interest (stress main effects, genotype main effect, and genotype-stress interaction) were extracted from the model using the *results()* function. Taxa with an FDR-adjusted *p*-value of less than 0.05 and a log_2_fold change > 2 were considered statistically significant.

ALDEx2 accounts for the compositional nature of count-relative abundance data uses the centred log-ratio (clr) transformation, which ensures the data are both scale invariant and sub-compositionally coherent. A model was constructed using the *aldex.glm()* function that handles complex study design using a model matrix of independent covariates. To examine our design factors individually, we constructed model testing for the average effect of genotype across all levels of stress via deviation coding of the stress variable (using the *contr.sum()* function) and vice versa. These results, as well as taxa effected by interaction between genotype and stress, were filtered for significance at a *p*-value < 0.05. Effect size could not be calculated due to the non-binary nature of some covariates.

To obtain consensus, these methods were compared based on which retain-resolved ASVs were considered significant for genotype effect, stress effect, and effect of interaction between genotype and stress status.

### 4.5. Microbe–Behaviour Correlation Analysis

To test for associations between specific behavioural traits and retain-resolved ASV abundance we implemented Microbiome Multivariable Association with Linear Models (*Maaslin2*) in R [70]. This method performs boosted additive general linear models to discover associations between metadata and the relative taxonomic abundances. Due to large compositional differences between B6 and *TCRβ-/-δ-/-* mice, this analysis was performed independently within each genotype group. To account for potentially confounding effects, we included sex and stress status as fixed effects in the model in addition to the behavioural outcome variables of interest. In addition to within-genotype analysis, omnibus analysis was also performed across all samples to assess microbe–behaviour correlations, modelling genotype as an additional fixed effect to account for microbiome variation between strains. Statistical significance was corrected for multiple testing using the Benjamini–Hochberg correction with significant associations identified at FDR < 0.1 and additional significance assigned to relationships with an FDR < 0.05.

## 5. Conclusions

Overall, our findings highlight the tripartite relationship between stress, microbe-immune interactions, and brain development. This study demonstrates the suitability of a novel behavioural pipeline for the study of genetic and environmental influences on neurodevelopment and illustrates the role of T cells in exacerbating the effects of early-life stress on emotional behaviour and gut microbiome structure. These data demonstrate that T-cell deficiency leads to the depletion of butyrate-producing bacteria (*Butyricicoccus*, *Oscillospira*, *Clostridia*, *Roseburia*) while allowing for the expansion of taxa that normally drive antibody-mediated immunity (*A. muciniphila*, *Muribaculaceae* uncharacterized taxa) as well as various species of *Lactobacillus*. Future work will investigate the role of specific taxa and T-cell subsets in mediating the interaction between microbes, immunity, and the environment and how these factors influence the neurodevelopmental trajectory of the host. Understanding these interactions between genetic risk factors and environmental exposures (including immune challenges and trauma) on brain development is instrumental to the development of microbiota-based therapeutic strategies aimed at fostering healthy maturation of the gut brain axis in neurodevelopmental disorders.

## Data Availability

The 16S rRNA gene sequencing and metabolomic data will be deposited to Brain-CODE, https://braininstitute.ca/research-data-sharing/brain-code, a secure neuroinformatics platform for data management, sharing, and analysis. While this study did not generate unreported custom codes, the codes used in the analysis are available on request from the authors.

## Supplementary Materials

The following supporting information can be downloaded at: https://www.mdpi.com/article/10.3390/ijms232315064/s1.
