# Supplementary material for: Microbe–Immune–Stress Interactions Impact Behaviour during Postnatal Development
Source: Int J Mol Sci. 2022 Dec 1;23(23):15064. doi: 10.3390/ijms232315064 (PMC9740388; doi:10.3390/ijms232315064)
Supplement: Supplementary file 1 [file ijms-23-15064-s001.zip › Supplemental_Material_2022_MG.pdf]

*Supplementary Material*

# Microbe-immune-stress interactions impact behaviour during postnatal development

Cassandra Francella[1], Miranda Green[1], Giorgia Caspani[2], Jonathan K.Y. Lai[1], Kelly C. Rilett[1], Jane A. Foster[1, 3]

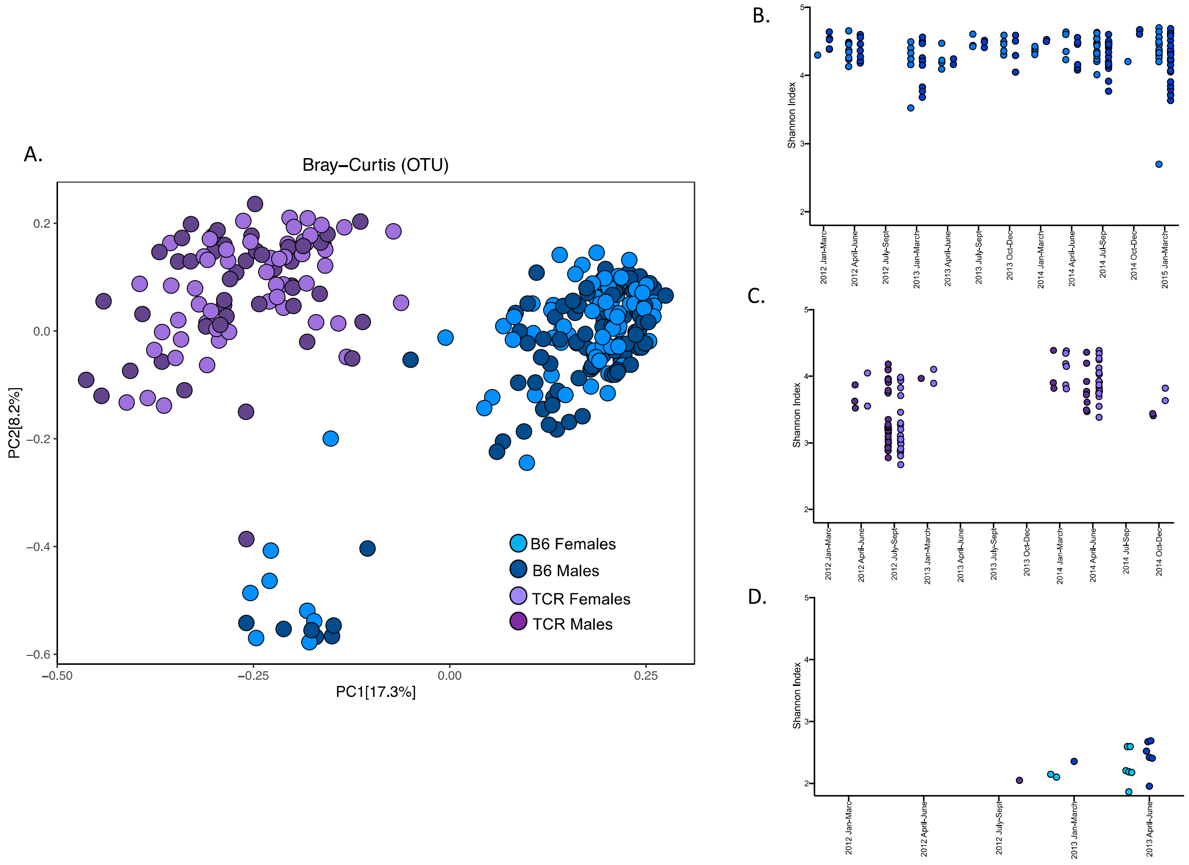


Supplemental Figure S1. Principle Component Analysis (PCoA) for Beta-diversity using raw ASV data revealed three individual clusters (A) including two B6 clusters (B, D) and one *TCRβ-/-δ-/-* cluster (C). Analysis of alpha diversity of individual samples showed that samples in the smaller B6 cluster (D) had lower alpha diversity compared to the larger B6 (B) and *TCRβ-/-δ-/-* cluster (C). The demographics of these samples revealed samples from the same litter as well as having similar postnatal days (Supplementary Table S1). Therefore, due to the nature of these samples, they were removed from subsequent analyses.

Supplemental Table S1 – Demographics for samples excluded from analyses

| Sample ID | Counts | Animal ID | LITTER | PO | GENOTYPE | SEX | Shannon Index |
|---|---|---|---|---|---|---|---|
| JF1419 | 2591 | D76-1 | D76 | 21-Feb-13 | B6 | F | 2.10 |
| JF1420 | 24724 | D76-2 | D76 | 21-Feb-13 | B6 | M | 2.36 |
| JF1470 | 59863 | D83-3 | D83 | 19-Apr-13 | B6 | M | 2.52 |
| JF1475 | 64361 | D84-1 | D84 | 01-May-13 | B6 | F | 1.87 |
| JF1486 | 64641 | D85-2 | D85 | 10-May-13 | B6 | M | 2.42 |
| JF1485 | 66697 | D85-1 | D85 | 10-May-13 | B6 | F | 2.60 |
| JF1303 | 66845 | D53-1 | D53 | 29-Aug-12 | TCR | M | 2.05 |
| JF1469 | 71810 | D83-2 | D83 | 19-Apr-13 | B6 | M | 2.67 |
| JF1478 | 74397 | D84-4 | D84 | 01-May-13 | B6 | M | 1.96 |
| JF1487 | 75488 | D85-3 | D85 | 10-May-13 | B6 | M | 2.41 |
| JF1482 | 76491 | D84-8 | D84 | 01-May-13 | B6 | F | 2.19 |
| JF1472 | 77097 | D83-5 | D83 | 19-Apr-13 | B6 | M | 2.69 |
| JF1483 | 80036 | D84-9 | D84 | 01-May-13 | B6 | F | 2.18 |
| JF1476 | 80101 | D84-2 | D84 | 01-May-13 | B6 | F | 2.21 |
| JF1473 | 80138 | D83-6 | D83 | 19-Apr-13 | B6 | F | 2.60 |
| JF1414 | 100261 | D75-5 | D75 | 21-Feb-13 | B6 | F | 2.15 |

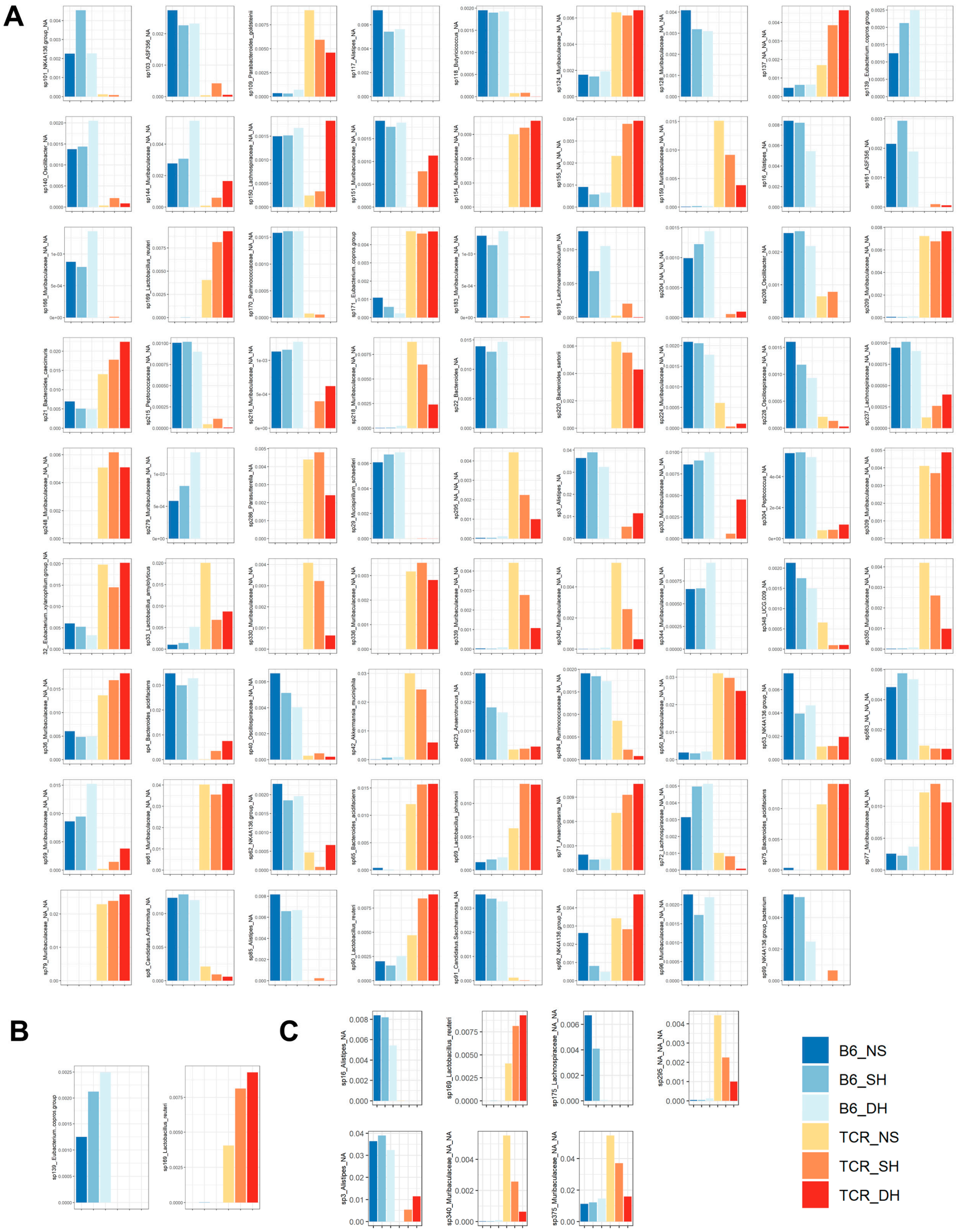
A
B
C
sp16_Alistipes_NA
sp169_Lactobacillus_reuteri
sp175_Lachnospiraceae_NA_NA
sp295_NA_NA_NA
sp3_Alistipes_NA
sp340_Muribaculaceae_NA_NA
sp375_Muribaculaceae_NA_NA
B6_NS
B6_SH
B6_DH
TCR_NS
TCR_SH
TCR_DH

Supplemental Figure S2. Relative abundance of significant consensus taxa from differential abundance analysis using ALDex2 and DESeq2 for (A) Genotype, (B) Stress and (C) Genotype-Stress interaction

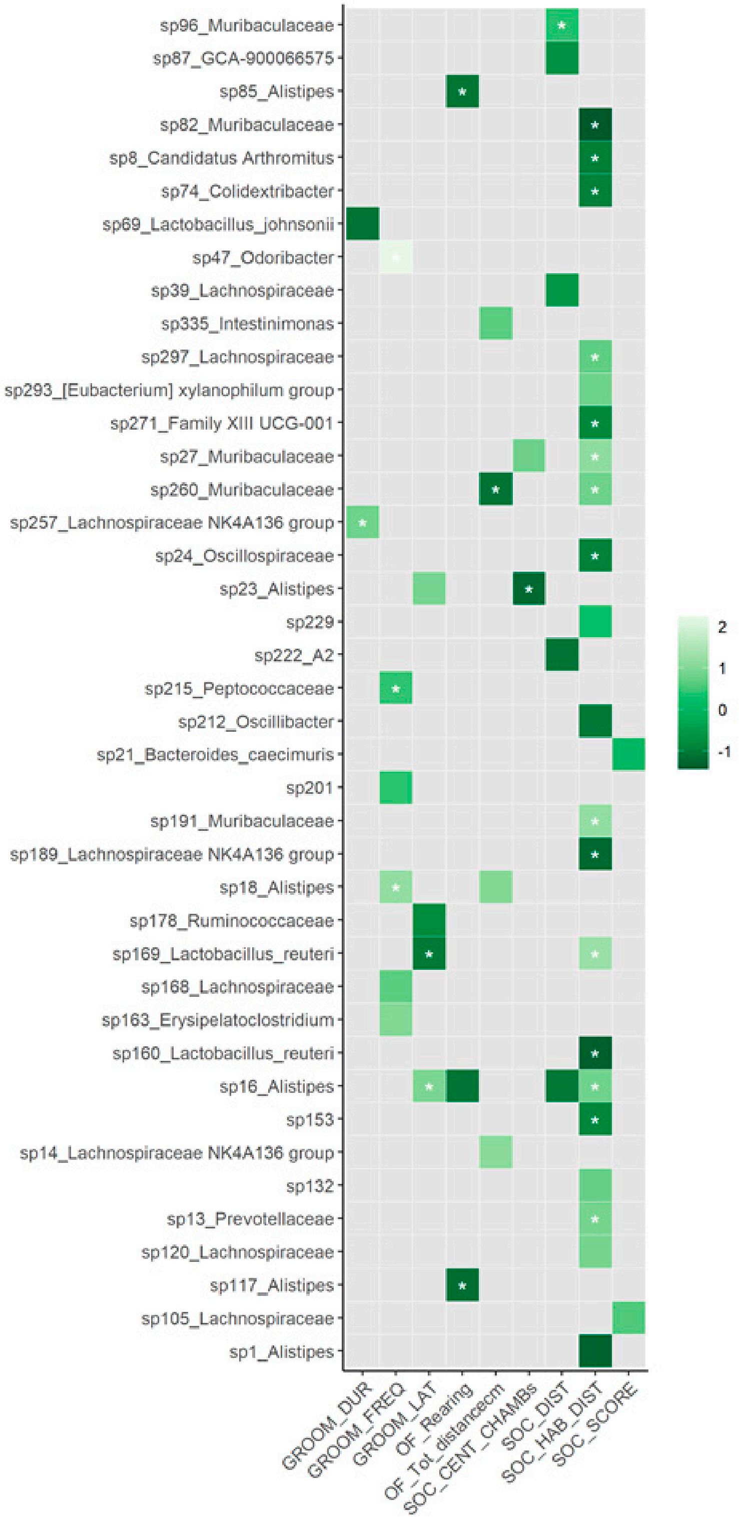
sp96_Muribaculaceae
sp87_GCA-900066575
sp85_Alistipes
sp82_Muribaculaceae
sp8_Candidatus Arthromitus
sp74_Colidextribacter
sp69_Lactobacillus_johnsonii
sp47_Odoribacter
sp39_Lachnospiraceae
sp335_Intestinimonas
sp297_Lachnospiraceae
sp293_[Eubacterium] xylanophilum group
sp271_Family XIII UCG-001
sp27_Muribaculaceae
sp260_Muribaculaceae
sp257_Lachnospiraceae NK4A136 group
sp24_Oscillospiraceae
sp23_Alistipes
sp229
sp222_A2
sp215_Peptococcaceae
sp212_Oscillibacter
sp21_Bacteroides_caecimuris
sp201
sp191_Muribaculaceae
sp189_Lachnospiraceae NK4A136 group
sp18_Alistipes
sp178_Ruminococcaceae
sp169_Lactobacillus_reuteri
sp168_Lachnospiraceae
sp163_Erysipelatoclostridium
sp160_Lactobacillus_reuteri
sp16_Alistipes
sp153
sp14_Lachnospiraceae NK4A136 group
sp132
sp13_Prevotellaceae
sp120_Lachnospiraceae
sp117_Alistipes
sp105_Lachnospiraceae
sp1_Alistipes
GROOM_DUR
GROOM_FREQ
GROOM_LAT
OF_Rearing
OF_Tot_distancecm
SOC_CENT_CHAMBs
SOC_DIST
SOC_HAB_DIST
SOC_SCORE
2
1
0
-1

Supplemental Figure S3. MaAsLin2 omnibus associations between behavioural measures (open field, social behaviour and self grooming) and gut microbiota composition at the ASV level in postnatal day 24 (P24) mice. Color scale-bar show scaled correlations between taxa and factors, ranging from the highest (white) to lowest (dark green). Stars indicate significance at FDR < 0.05.
